# Statistical Fragility of Surgical and Procedural Clinical Trials in Orthopaedic Oncology

**DOI:** 10.5435/JAAOSGlobal-D-19-00152

**Published:** 2020-06-01

**Authors:** Lynn Ann Forrester, Eugene Jang, Michelle M. Lawson, Ana Capi, Wakenda K. Tyler

**Affiliations:** From the Department of Orthopedic Surgery, Columbia University Medical Center, New York, NY (Dr. Forrester, Dr. Jang, Ms. Capi, and Dr. Tyler) and the Department of Orthopaedics, University of Rochester, Rochester, NY (Ms. Lawson).

## Abstract

The fragility index (FI) is a powerful tool that can be used to assess the statistical strength of a study outcome. This metric is defined as the number of patients who would need to have an alternative outcome to convert a clinical trial result from statistically significant to not statistically significant, or vice versa. No studies to date have used the FI to evaluate surgical and procedural clinical trials in the orthopaedic oncology literature. The primary purpose of this study was to use the FI to evaluate the statistical strength of widely cited surgical and procedural clinical trials in orthopaedic oncology.

**Methods:**

We performed a PubMed search for orthopaedic oncology clinical trials in high impact orthopaedics–focused, oncology-focused, and general medicine journals. For each study included in this analysis, we calculated the FI for all identified dichotomous, categorical outcomes.

**Results:**

We identified 23 studies with 48 outcomes. Twelve of these outcomes were statistically significant, with a median FI of two. Nine studies addressed the number of patients lost to follow up, and the FI was less than the number of patients lost to follow up for most outcomes (60%) in these studies.

**Conclusions:**

The orthopaedic oncology literature has substantial statistical fragility, likely explained by a high number of patients lost to follow up and small sample sizes. More multicenter, cooperative studies are necessary to increases the robustness of clinical research in orthopaedic oncology.

The *P* value is a powerful statistical tool that is commonly used to evaluate outcomes in research. However, the *P* value exclusively provides information relevant to the compatibility of data with a null hypothesis while providing no information concerning effect size, strength of association, or applicability of a research outcome to a specific population.^1^ Recently, both researchers and statisticians have advocated for lowering *P* value thresholds, reporting exact *P* values, or even abandoning *P* values completely in an effort to improve the critical evaluation of research outcomes.^1,2^ Walsh et al and other research groups have advocated for the use of alternative measures of statistical association such as the fragility index (FI) to act as a partner to the *P* value.^3,4,5,6,7,8,9,10,11,12,13,14^

The FI is calculated by step-wise altering the outcome status of patients included in one study arm, with the goal of determining how many event changes would be necessary to switch the outcome from statistically significant (*P* < 0.05) to not statistically significant (*P* > 0.05), or vice versa. A large FI suggests that many events would need to change to alter the original observed result, giving the reader more confidence in the statistical strength of the study outcome.

The FI for orthopaedic subspecialties is generally low, with reported FIs ranging from two to five.^3,5,6,11,13^ Thus far, no studies have used the FI to evaluate the musculoskeletal oncology literature. The primary objective of this study was to use the FI to evaluate the statistical strength of widely cited surgical and procedural studies in the orthopaedic oncology literature. A secondary goal for this study was to identify features of clinical trials that are associated with greater statistical fragility.

## Methods

### Study Design and Eligibility Criteria

We performed a systematic survey of clinical trials in musculoskeletal oncology published in high-impact journals. First, we identified the highest impact journals relevant to orthopaedic oncology. Using InCites Journal Citation Reports, we performed three searches in a manner similar to previous work evaluating statistical fragility in healthcare research.^4,12,13^ The first search identified the top 50 highest impact orthopaedic journals (journal group 1) and the second search identified the top 50 highest impact oncology journals (journal group 2). In the third search, we screened the top 100 highest impact science journals. After eliminating journals that were previously identified in the first two searches and excluding journals without a focus in biology or medicine, we identified 58 additional high-impact medicine journals (journal group 3).

Next, we performed three searches in PubMed for clinical trials published in journals included in each of the abovementioned journal groups. Our search included studies published between January 1, 1990, and December 31, 2018. For journal group 1, we also applied the medical subject heading major topic “neoplasms” to identify oncology studies in the orthopaedics literature. For journal groups 2 and 3, we applied the medical subject heading major topic “musculoskeletal diseases” to identify studies relevant to orthopaedics in the oncology and medicine literature.

After performing each of the searches discussed above, we screened all titles for relevance to orthopaedic oncology and all remaining abstracts for surgical or procedural interventions. Finally, as previously described in the study by Walsh et al,^14^ we read each of the remaining studies and identified all dichotomous, categorical study outcomes that could be appropriately described using 2 × 2 contingency tables.

### Study Characteristics

We collected the following information from each study that met the inclusion criteria: title, publication year, use of randomization, patient sample size, number of patients lost to follow up, study outcomes, reported *P* value, and journal title. Then, we filled out a 2 × 2 contingency table for each dichotomous, categorical study outcome. Next, we used the InCites Journal Citation Reports to identify the journal impact factor and number of journal citations, and the National Institutes of Health iCite database to identify the relative citation ratio (RCR) for each of the studies included in this analysis.^15-17^ Finally, we used the Web of Science to collect data on the number of citations for each of the studies evaluated in our study.^18^

### Calculation of Fragility Index

Using the method previously described by Walsh et al,^14^ we calculated the FI for all categorical, dichotomous outcomes reported in the studies included in this study. First, we recalculated the *P* value for each outcome using the Fisher exact test. In all studies, the significance of the recalculated *P* value matched the significance described in the study. Then, we identified the intervention group with the smallest number of events. If the recalculated *P* value was statistically significant, we switched events from one outcome to another, step-wise, until the calculated *P* value was greater than 0.05. The smallest change in the number of outcomes that was sufficient to obtain a *P* value greater than 0.05 was calculated as the FI for that outcome. Conversely, if the recalculated *P* value was not statistically significant, we performed the same process until the calculated *P* value was less than 0.05.

### Statistical Analysis

We used descriptive statistics to evaluate the outcomes included in this study. We also used the Pearson correlation coefficient to determine associations between study variables and the Student *t*-test to characterize differences between subpopulations of the study data. All analyses were performed using Microsoft Excel (2007) and SPSS (Version 19.0).

Given that multiple outcomes were identified per study, we were concerned that including all FIs in all correlation calculations would inappropriately weight studies with a higher number of outcomes compared with studies with a lower number of outcomes. Thus, we used the highest calculated FI from each study in all calculations comparing publication-level variables. Publication-level variables included patient sample size, RCR, publication year, number of article citations, journal impact factor, and number of journal citations.

## Results

### Study Selection

We identified 162 and 506 articles in our searches using journal groups 1 and 2, respectively. We screened these 668 titles and excluded studies that did not examine common pathologies seen by orthopaedic oncologists. Examples of excluded topics included bursitis, Dupuytren contracture, and Morton neuroma. Then, we screened the remaining 475 abstracts for surgical or procedural interventions and excluded studies studying exclusively chemotherapy- or radiotherapy-based interventions from further review. Next, we read the remaining 137 articles to identify any dichotomous, categorical outcomes that could be evaluated using 2 × 2 contingency tables. At the conclusion of this screening process, we selected 23 articles for further evaluation. When searching PubMed using journal group 3, we initially identified an additional 21 articles. However, after screening all of these titles for relevance to orthopaedic oncology, we did not include any of these articles in further analyses.

### Characteristics of Trials and Outcomes

The 23 identified studies were published between 1991 and 2017. Eight of the reviewed studies were published before the year 2000, eight were published between the years 2000 and 2009, and seven were published during or after 2010. Studies were published in the following orthopaedics-focused journals: *Clinical Orthopaedics and Related Research*, *Orthopaedics*, *Clinical Spine Surgery*, *European Spine Journal*, *International Orthopaedics*, *Journal of Bone and Joint Surgery*, *Journal of Hand Surgery*, *Journal of Spinal Disorders and Techniques*, and *Spine* (Table 1). Studies were also published in the following oncology-focused journals: *Cancer*, *Journal of Clinical Oncology*, and the *Annals of Oncology* (Table 2). Overall, 14 of 23 studies (61%) were published in orthopaedics-focused journals, and 9 of 23 studies (39%) were published in oncology-focused journals. Seven of the 23 evaluated studies (30%) used randomization to allocate patients into intervention groups. The remaining studies were primarily either retrospective or allocated patients to treatment groups according to patient preference.

**Table 1:** Studies Published in Orthopaedics-focused Journals

Orthopaedics-focused Journal	No. of Publications
*Clinical Orthopaedics and Related Research*	5
*Orthopaedics*	2
*Clinical Spine Surgery*	1
*European Spine Journal*	1
*International Orthopaedics*	1
*Journal of Bone and Joint Surgery*	1
*Journal of Hand Surgery*	1
*Journal of Spinal Disorders and Techniques*	1
*Spine*	1

**Table 2:** Studies Published in Oncology-focused Journals

Oncology-focused Journal	No. of Publications
*Cancer*	6
*Journal of Clinical Oncology*	3
*Annals of Oncology*	1

We identified 48 outcomes in the 23 studies discussed above. Fourteen of the 48 outcomes (29%) were primary outcomes, and 34 of the 48 outcomes (71%) were secondary outcomes. Trials reported outcomes that could be exclusively placed in one of the following categories: postoperative complications (29%), survival (25%), patient pain and/or function (15%), radiographic findings (8.3%), tumor recurrence (8.3%), surgical margins (8.3%), disease progression (4.2%), or histopathological outcomes (2.1%). The 23 trials examined in this study had a median sample size of 67 patients (mean 81, range 10 to 355), and the median number of patients lost to follow up per outcome was 3.0 (mean 9.2, range 0 to 44). The median journal impact factor was 4.09 (mean 7.28, range 1.46 to 26.36), and the median journal citation number was 40,313 (mean 51,964, range 406 to 156,476).

### Fragility Index

The median FI for all 48 outcomes included in this analysis was 4 (mean 6, range 1 to 92). Of the studied outcomes, 12 were statistically significant and 36 were not statistically significant. The median FI for statistically significant outcomes was 2 (mean 11, range 1 to 92), and the median FI for outcomes that were not statistically significant was 4 (mean 4, range 1 to 9). No statistically significant difference were noted between the FIs calculated for significant and nonsignificant results (*P* = 0.114).

No correlation existed between FI and initial, recalculated *P* values (R = −0.076, *P* = 0.608). However, when exclusively examining studies with statistically nonsignificant outcomes, a strong positive correlation was observed between FI and reported *P* value (R = 0.7399, *P* < 0.0001). Comparable findings were not observed for studies with outcomes that were statistically significant (R = −0.377, *P* = 0.227).

The FI was less than or equal to 3 events in 21 of the 48 reviewed study outcomes (44%). Only 9 of the 23 evaluated studies reported whether they lost patients to follow up (39%). Twenty outcomes were reported in these 9 articles, and the FI was less than or equal to the total number of patients lost to follow up for 12 of those 20 outcomes (60%). No statistically significant association was observed between number of patients lost to follow up and FI (R = 0.181, *P* = 0.446).

When evaluating publication-level variables, we found that FI was strongly correlated with patient sample size (R = 0.840, *P* < 0.00001). However, no statistically significant associations were observed between FI and the following study variables: RCR, publication year, number of article citations, journal impact factor, and number of journal citations (Table 3).

**Table 3:** Publication-level Associations Between Fragility Index and Study Variables

Study Variables	Pearson Correlation Coefficient	*P* Value
Patient sample size	0.846	<0.001
RCR	0.321	0.179
Publication year	−0.365	0.087
No. of article citations	0.0430	0.850
Journal impact factor	0.192	0.380
No. of journal citations	−0.035	0.878

RCR = relative citation ratio

The median FI of outcomes reported in the articles published in orthopaedics-focused journals was three (mean 3, range 1 to 9), and the median FI of outcomes reported in articles published in oncology-focused journals was six (mean 11, range 1 to 92). No statistically significant difference existed in the FIs for outcomes reported in the articles from each type of journal (*P* = 0.070). No statistically significant difference was noted in the patient sample size between studies published in orthopaedics- and oncology-focused journals (*P* = 0.145).

The number of times an article was cited was strongly correlated with journal impact factor (R = 0.694, *P* < 0.001). However, patient sample size was not correlated with number of citations (R = 0.0120, *P* = 0.957) or journal impact factor (R = 0.235, *P* = 0.280). In addition, there was no association between RCR and publication year (R = −0.283, *P* = 0.242), confirming that RCR accounts for time in circulation when reporting the scientific influence of an article.^17^

## Discussion

Randomized controlled trials in the field of orthopaedic oncology are relatively rare, as with many other orthopaedic subspecialties. In this study, we identified 23 clinical trials examining procedural and surgical interventions in orthopaedic oncology that have been published in the past 28 years. These findings suggest that there is a relative paucity of studies evaluating surgical and procedural interventions in orthopaedic oncology. In addition, only seven of these trials used randomization to allocate patients into treatment groups, suggesting that relatively few prospective, randomized clinical trials are present in orthopaedic oncology. A potential explanation for these findings is that approximately 2,700 bone and 5,700 soft-tissue sarcomas are diagnosed each year in the United States, representing <1% of all malignancies.^19^ Given that orthopaedic oncologists treat an average of 20 bone and 38 soft-tissue sarcomas annually, a relatively small study population may slow the building of clinical trials or limit potential sample size of studied interventions.^20^

### Key Findings

This is the first study to examine the FI for surgical and procedural clinical trials in orthopaedic oncology. We found that the median FI for all outcomes evaluated in this study was four. We also found that the median FI was two for statistically significant outcomes and four for outcomes that were not statistically significant. Some previous FI studies have exclusively reviewed statistically significant results; however, given that clinical practice guidelines are also based on null results, we believed that it was appropriate to evaluate the outcomes that were both statistically and not statistically significant.

Despite the relative rarity of the conditions treated by orthopaedic oncologists, we found the statistical fragility of the musculoskeletal oncology literature to be comparable with other orthopaedic subspecialties.^3,5,6,11,13^ Other surgical subspecialties also have comparable FIs with orthopaedic subspecialties, with otolaryngology reported to have a FI of one and urology reported to have a FI of three.^9,10^ However, studies examining statistical fragility in general medicine and pediatrics have reported FIs of eight and seven, respectively.^7,14^ In addition, Checketts et al^21^ recently found that studies informing the American Academy of Orthopaedic Surgeons Clinical Practice Guidelines that are listed as having “strong evidence” have a median FI of two. These findings suggest that there is still substantial room for improvement in not only study quality but also evaluation of study outcomes in orthopaedic oncology.

Our secondary goal of this study was to identify study characteristics that are associated with increased statistical fragility. We observed a strong positive correlation between FI and patient sample size, but observed no associations between FI and other study characteristics. Musculoskeletal oncologists have recently placed greater emphasis on collaboration among institutions and have prioritized efforts to reach a consensus on research questions.^19^ These efforts are likely to facilitate increased patient sample size in future clinical trials, and ideally improve the quality of research in orthopaedic oncology.

### Strengths and Limitations

The primary strength of this study was the methodology that we used to search for clinical trials in orthopaedic oncology. Musculoskeletal oncology is a multidisciplinary field involving collaborations with myriad other medical fields; thus, we searched high-impact orthopaedics, oncology, and medical journals. If we had used a search methodology equivalent to previous studies evaluating statistical fragility in orthopaedic subspecialties, we would have missed the 10 studies that we identified in oncology-focused journals. This more rigorous approach to evaluating the orthopaedic oncology literature was necessary to maximize the number of studies included in this analysis. One shortcoming to the FI as a statistical tool is that we can only use it to evaluate outcomes with categorical, dichotomous variables. Hence, the relatively limited application of this statistical tool limits the applicability of the findings of our study to the orthopaedic oncology literature as a whole.

## Conclusions

The FI serves as an intuitive tool that orthopaedic surgeons can use to evaluate the statistical strength of research outcomes. Appropriate application of this tool is likely to facilitate more rigorous interpretation of clinical trial findings. The orthopaedic oncology literature exhibits a relative paucity of prospective, randomized clinical trials and substantial statistical fragility, suggesting there is more work to be done to improve research quality in the field. Our study found a strong correlation between FI and patient sample size, suggesting that continued support of collaborative, multicenter trials in orthopaedic oncology are likely to strengthen the quality of clinical trials, and ideally facilitate improved quality of patient care.
